# Supplementary material for: Physicians’ perspective on potentially non-beneficial treatment when assessing patients with advanced disease for ICU admission: a qualitative study
Source: BMJ Open. 2021 May 21;11(5):e046268. doi: 10.1136/bmjopen-2020-046268 (PMC8144032; doi:10.1136/bmjopen-2020-046268)
Supplement: Supplementary data [file bmjopen-2020-046268supp001.pdf]

Supplementary file: Interview guide

Please tell me about the first / second situation you have chosen to discuss today.

Prompts:

- Patient characteristics: age? underlying illness? goals of care?
- Context: when dit it occur? what was the reason for calling the ICU? was the patient admitted to the ICU?
- Interactions between the internal medicine and the intensive care physicians:
    - Did the ICU physician come to see the patient?
    - Did you know the other physician?
    - Was the other physician senior or junior to you?
    - What were your expectations with respect to the other physician?

In your opinion, what made the decision-making process easier or more difficult in this situation?

Could you compare the two situations? (after the participant had discussed the two situations)

In your opinion, what is an ideal ICU admission decision-making process?

Escher M, *et al. BMJ Open* 2021; 11:e046268. doi: 10.1136/bmjopen-2020-046268
