# Supplementary material for: Physicians’ perspective on potentially non-beneficial treatment when assessing patients with advanced disease for ICU admission: a qualitative study
Source: BMJ Open. 2021 May 21;11(5):e046268. doi: 10.1136/bmjopen-2020-046268 (PMC8144032; doi:10.1136/bmjopen-2020-046268)
Supplement: Author's manuscript [file bmjopen-2020-046268.draft_revisions.pdf]

BMJ Open is committed to open peer review. As part of this commitment we make the peer review history of every article we publish publicly available.

When an article is published we post the peer reviewers' comments and the authors' responses online. We also post the versions of the paper that were used during peer review. These are the versions that the peer review comments apply to.

The versions of the paper that follow are the versions that were submitted during the peer review process. They are not the versions of record or the final published versions. They should not be cited or distributed as the published version of this manuscript.

BMJ Open is an open access journal and the full, final, typeset and author-corrected version of record of the manuscript is available on our site with no access controls, subscription charges or pay-per-view fees (http://bmjopen.bmj.com).

If you have any questions on BMJ Open's open peer review process please email
info.bmjopen@bmj.com

BMJ Open

Physicians' perspective on nonbeneficial treatment when assessing patients with advanced disease for ICU admission: a qualitative study.

Journal:	*BMJ Open*
Manuscript ID	bmjopen-2020-046268
Article Type:	Original research
Date Submitted by the Author:	01-Nov-2020
Complete List of Authors:	Escher, Monica; University Hospitals Geneva, Division of Palliative Medicine; University of Geneva Faculty of Medicine, Unit for Development and Research in Medical Education (UDREM)<br>Nendaz, Mathieu; University of Geneva Faculty of Medicine, Unit of Development and Research in Medical Education (UDREM); University Hospitals Geneva, Division of General Internal Medicine<br>Cullati, Stéphane; University Hospitals Geneva, Quality of Care Service; University of Fribourg Faculty of Science and Medicine, Population Health Laboratory<br>Perneger, Thomas; University Hospitals Geneva, Division of Clinical Epidemiology<br>Hudelson, Patricia; University Hospitals Geneva, Department of Primary Care
Keywords:	INTENSIVE & CRITICAL CARE, MEDICAL ETHICS, PALLIATIVE CARE, QUALITATIVE RESEARCH

SCHOLARONE™
Manuscripts

I, the Submitting Author has the right to grant and does grant on behalf of all authors of the Work (as defined in the below author licence), an exclusive licence and/or a non-exclusive licence for contributions from authors who are: i) UK Crown employees; ii) where BMJ has agreed a CC-BY licence shall apply, and/or iii) in accordance with the terms applicable for US Federal Government officers or employees acting as part of their official duties; on a worldwide, perpetual, irrevocable, royalty-free basis to BMJ Publishing Group Ltd ("BMJ") its licensees and where the relevant Journal is co-owned by BMJ to the co-owners of the Journal, to publish the Work in this journal and any other BMJ products and to exploit all rights, as set out in our licence.

The Submitting Author accepts and understands that any supply made under these terms is made by BMJ to the Submitting Author unless you are acting as an employee on behalf of your employer or a postgraduate student of an affiliated institution which is paying any applicable article publishing charge ("APC") for Open Access articles. Where the Submitting Author wishes to make the Work available on an Open Access basis (and intends to pay the relevant APC), the terms of reuse of such Open Access shall be governed by a Creative Commons licence – details of these licences and which Creative Commons licence will apply to this Work are set out in our licence referred to above.

Other than as permitted in any relevant BMJ Author's Self Archiving Policies, I confirm this Work has not been accepted for publication elsewhere, is not being considered for publication elsewhere and does not duplicate material already published. I confirm all authors consent to publication of this Work and authorise the granting of this licence.

For peer review only

Physicians' perspective on nonbeneficial treatment when assessing patients with advanced disease for ICU admission: a qualitative study.

Monica Escher,[1,2] Mathieu Nendaz,[2,3] Stéphane Cullati,[1,4,5] Thomas Perneger,[6] Patricia Hudelson.[7]

1. Division of Palliative Medicine, Geneva University Hospitals, Geneva, Switzerland

2. Unit of Development and Research in Medical Education, Faculty of Medicine, University of Geneva, Geneva, Switzerland

3. Division of General Internal Medicine, Geneva University Hospitals, Geneva, Switzerland

4. Quality of Care Service, Geneva University Hospitals, Geneva, Switzerland

5. Population Health Laboratory (#PopHealthLab), Faculty of Science and Medicine, University of Fribourg, Fribourg, Switzerland

6. Division of Clinical Epidemiology, Geneva University Hospitals, Geneva, Switzerland

7. Division of Primary Care Medicine, Geneva University Hospitals, Switzerland

Corresponding author:

Monica Escher

Division of Palliative Medicine

Geneva University Hospitals

Rue Gabrielle-Perret-Gentil 4

1211 Geneva - Switzerland

E-mail address: monica.escher@hcuge.ch

Telephone number: +41 22 372 29 23

For peer review only

For peer review only - http://bmjopen.bmj.com/site/about/guidelines.xhtml

Word count

Abstract: 234

Text: 1536

Figure: 1

References: 14

For peer review only

For peer review only - http://bmjopen.bmj.com/site/about/guidelines.xhtml

Abstract

Objective: The use of intensive care at the end of life can be high, leading to inappropriate healthcare utilization, and prolonged suffering for patients and families. The objective of the study was to determine which factors influence physicians' admission decisions in situations of potentially nonbeneficial intensive care.

Design: This is a secondary analysis of a qualitative study exploring the triage process. In-depth interviews were analysed using an inductive approach to thematic content analysis.

Setting: Data were collected in a Swiss tertiary care center between March and June 2013.

Participants: 12 ICU physicians and 12 internists routinely involved in ICU admission decisions.

Results: Physicians struggled to understand the request for intensive care for patients with advanced disease and full code status. Physicians considered patients' long-term vital and functional prognosis, but they also resorted to shortcuts, i.e. a priori consensus about reasons for admitting a patient. Family pressure and unexpected critical events were determinants of admission to the ICU. Patient preferences, ICU physician's expertise and collaborative decision making facilitated refusal. Physicians were willing to admit a patient with advanced disease for a limited amount of time to fulfill a personal need.

Conclusions: In situations of potentially nonbeneficial intensive care, the influence of shortcuts or context-related factors suggests that practice variations and inappropriate admission decisions are likely to occur. Institutional guidelines and timely goals of care discussions with patients with advanced disease and their families could contribute to ensuring appropriate levels of care.

For peer review only

Strengths and limitations of this study

- Participant sample was representative of physicians involved in ICU admission decisions in our institution.
- In-depth interviews were conducted by an experienced medical sociologist.
- Data analysis was done by a multidisciplinary research team including clinicians from the intensive care, internal medicine, and palliative care fields, a medical sociologist, and a medical anthropologist.
- The main limitation of this study is that it is a secondary analysis of interviews that did not specifically focus on the role of nonbeneficial treatment in ICU admission decisions.

For peer review only

Keywords: intensive care, medical futility, decision-making, palliative care, end of life care

Funding

This work was supported by the Swiss National Science Foundation, National Research Program "End of Life" (NRP 67) grant number 139304.

Competing interests statement

The authors declare no potential conflicts of interest with respect to the research, authorship, and/or publication of this article.

Data availability statement

All data relevant to the study are included in the article or uploaded as supplementary information. No additional data are available.

For peer review only

INTRODUCTION

The use of intensive care in the last month of life can be high, especially for non cancer patients.[1] Providing nonbeneficial treatments to patients with advanced disease only prolongs suffering at the end of life. It is associated with family distress[2] and healthcare staff burnout[3]. Nonbeneficial care is a concern for patients admitted to the intensive care unit (ICU),[4, 5] but it may be an issue during triage. Deciding whether to admit patients with advanced disease to the ICU is often complex, and physicians mostly rely on their clinical judgment.[6] A particularly difficult situation involves critically ill patients with advanced disease for whom physicians consider limiting treatment intensity, and who have a full code status.[7] We aimed to determine whether physicians integrate potentially nonbeneficial treatments in their reasoning for ICU admission decisions and how they resolve the question.

METHODS

This is a secondary analysis of a qualitative study exploring the triage process.[7] The study was conducted at a tertiary care hospital. It was approved by the Geneva Research Ethics Committee.

Participants and data collection

Physicians working in the Divisions of General Internal Medicine and of Intensive Care, and routinely assessing patients for intensive care were eligible. We used a convenience sampling strategy, including similar numbers of internists (n=12) and ICU physicians (n=12). Internists were certified chief residents (n=8) and residents (n=4), as they are involved in admission decisions during night calls. ICU physicians were chief residents (n=7) and attendings (n=5). Participants were recruited between March and June 2013 after

For peer review only - http://bmjopen.bmj.com/site/about/guidelines.xhtml

presentations of the study at staff meetings and through invitations sent by email. They gave written consent.

The interview guide was pre-tested with two internists and two ICU physicians (Supplementary file). A medical sociologist, who had neither previous nor hierarchical relationship to interviewees, conducted in-depth qualitative interviews. Participants were asked to reflect on their experience of two significant ICU admission decisions involving a medical inpatient. The main objective of the study was to determine the factors facilitating or hindering admission decisions. Interviews lasted 57 minutes on average (min 26, max 94). They were recorded, transcribed *verbatim* and anonymised.

Analysis

Interview transcripts were analysed using an inductive approach to thematic content analysis [8]. Four interviews (two with internists, two with ICU physicians) were independently read by members of the multidisciplinary group. Key themes and emergent ideas related to potentially nonbeneficial intensive care ("medical futility") were identified. A list of codes was developed and used to independently double-code the first 4 interviews. Coding discrepancies were resolved by consensus. A third researcher cross-checked the coded interviews and the list of codes was updated. The other interviews were coded by one researcher and cross-checked by two researchers. New codes were continuously created until saturation, and were used to recode previous interviews. Codes were clustered according to their content relatedness (e.g. "intensive care as default option"). Coding and analysis were conducted using Atlas.ti Scientific Software Development (Version 7.0.71).

Patient and public involvement

No patients were involved.

RESULTS

For peer review only - http://bmjopen.bmj.com/site/about/guidelines.xhtml

Participant characteristics

Among the 24 physicians, 17 were male. Mean age was 38 years (range 27-51) and mean number of years since graduation was 11.8 (SD 6.8). On average ICU physicians were older and more experienced than internists. Three internal medicine residents had never worked in an ICU.

Clinical situations during triage

Physicians described two scenarios, when the decision to admit or refuse a patient to intensive care was straightforward (Figure). Either there was a medical indication, i.e. short-term benefit, and high intensity care was considered appropriate and was congruent with code status, then the patient was admitted; or there was no medical indication, and then the patient was refused.

In situations where there was no medical indication, physicians explained that sometimes context-related factors, i.e. social pressure due to a patient's prominence, and concerns about patient's safety on the ward, could lead to the patient being admitted.

> *"Probably the patient would not have had any benefit from intensive care, … but sometimes we must admit [a patient], precisely when there is some doubt, because we choose the safe side." (ICU12)*

Concerns about nonbeneficial treatment arose mainly for patients with advanced disease, for whom high intensity care seemed inappropriate but who had a full code status (scenario II). In these situations, physicians struggled to understand the request for treatment.

> *"When the patient has a cancer at a very advanced stage and still, it is decided to intubate him because he has a pulmonary infection, is an admission to intensive care really meaningful?" (ICU01)*

For peer review only - http://bmjopen.bmj.com/site/about/guidelines.xhtml

Factors influencing ICU admission decision in the case of potentially nonbeneficial treatments

Participants described factors that oriented a decision towards admission, towards refusal, or that were used for either decision (Figure). There was consensus among respondents that intensive care should be provided as a default option in cases of great uncertainty, for patients needing intensive care as a consequence of an iatrogenic event, or for patients with onco-hematological diseases.

"When in doubt, we admit and we treat." (ICU10)

"When there are so-called iatrogenic complications, I feel I have a responsibility to treat the complication, … to make abstraction of the patient's general context and to use all available means to take care of it." (MED11)

In addition, respondents reported that they could be pressured into admitting a patient with advanced disease by the family or the referring physician. Factors related to the acute event could also prompt physicians to admit a patient for whom limited treatment intensity had previously been decided.

"Some families demand everything, even though it is futile,… and they put an enormous pressure on the system." (ICU04)

Physicians were also willing to provide life-sustaining treatments to a terminally ill patient for a limited amount of time in order to fulfill a personal need of the patient or family.

"Even in a desperate situation, we can admit a patient to intensive care if we know there is something coming up; we wait for a relative who is on his way" (ICU11)

Determinants of ICU refusal in the case of nonbeneficial treatment involved not only consideration of patient preferences but were also influenced by professional interactions.

For peer review only - http://bmjopen.bmj.com/site/about/guidelines.xhtml

The ICU physician's expertise carried weight; collaborative decision making between internists and ICU physicians facilitated refusal as did physicians' recognition that ad hoc evaluation was at times as valuable as code status.

"We decided to go against the code status. But we did it together, we evaluated the patient, we discussed" (MED01)

In such cases, and depending on the type of the acute event, the patient could be admitted or not. Physicians also took into account long term prognosis. They considered patient-related factors, i.e. age, comorbidities, functional status and quality of life, and disease-related factors, i.e. prognosis, and availability of disease-directed treatments.

DISCUSSION

Physicians in our study gave consideration to the role of potentially nonbeneficial treatment in the admission decision for patients with advanced disease in situations of uncertainty. They took many factors into account, which reflects how complex the decision may be. They reasoned about patient's long-term prognosis, but they also resorted to shortcuts, i.e. a priori consensus about reasons for admitting a patient. Human factors influence the decision towards admission: physicians felt pressure on the part of the family and as a consequence of unexpected critical events. Physicians' response to unexpected events could be ethically problematic and lead to potentially inappropriate admissions to intensive care. A recent study has shown that patients are willing to trade survival time to avoid end of life in an ICU.[9] As to the opinion of the family, it has been shown to significantly influence ICU admission decisions.[7, 10, 11] Family can either act as useful healthcare surrogates or make requests in response to their own needs[12, 13].

ICU physicians' expertise and collaborative decision making are factors that can facilitate a decision not to admit a patient. Such decisions are difficult to make and

For peer review only - http://bmjopen.bmj.com/site/about/guidelines.xhtml

physicians may be willing to admit patients to the ICU so that they or their family can fulfill a personal need. Time-limited trial is an accepted strategy for patients with a poor prognosis when survival benefit with intensive care or patient preferences are unclear, or when patient and/or family need time to adapt.[14] Such an approach is concordant with the intention to provide patient- and family-centered sensitive care.

Our study has limitations. It is a secondary analysis of interviews that did not specifically focus on the role of nonbeneficial treatment in ICU admission decision making. Other issues might arise in a more in-depth study on this topic. In addition, the study was conducted in a context where internists and ICU physicians collaborate when deciding on ICU admission. Where this is not the case, physicians may be influenced by different factors. Nonetheless, we were able to identify several factors that physicians considered when deciding on ICU admission when treatment was deemed nonbeneficial.

CONCLUSION

Physicians are concerned about providing nonbeneficial intensive care treatment for critically ill patients with advanced disease in situations of uncertainty. The ICU admission decision is then complex and influenced by a variety of medical and contextual factors. The role that shortcuts or context-related factors may play raises concerns about potentially inappropriate admission to intensive care. Our results highlight the risk of practice variation in ICU admission decisions. Additional research should focus on how physicians weigh multiple contextual factors, and on how institutional guidelines and advance care planning with patients and families can help admission decisions and contribute to ensuring appropriate levels of care.

For peer review only

For peer review only - http://bmjopen.bmj.com/site/about/guidelines.xhtml

Author contributions

M.E. and T.P. contributed to study concepts. M.E., M.N, S.C., T.P., P.H. contributed to study design. S.C. and M.E. collected the data. All the authors contributed to quality control of the data. All authors contributed to data analysis and interpretation. M.E. drafted the manuscript. All authors contributed to manuscript editing and review.

Acknowledgments

The authors thank the physicians who participated in the study. We also thank Prof Bara Ricou for her contribution to the whole project about the determination of factors influencing ICU admission decisions.

For peer review only

REFERENCES

1. Teno JM, Gozalo P, Trivedi AN et al. Site of death, place of care, and health care transitions among US Medicare beneficiaries, 2000-2015. *JAMA* 2018;320:264-271.
2. Chen C, Michaels J, Meeker MA. Family outcomes and perceptions of end-of-life care in the intensive care unit: a mixed-methods review. *J Palliat Care* 2019:825859719874767.
3. Schwarzkopf D, Ruddel H, Thomas-Ruddel DO et al. Perceived nonbeneficial treatment of patients, burnout, and intention to leave the job among ICU nurses and junior and senior physicians. *Crit Care Med* 2017;45:e265-e273.
4. Piers RD, Azoulay E, Ricou B et al. Perceptions of appropriateness of care among European and Israeli intensive care unit nurses and physicians. *JAMA* 2011;306:2694-2703.
5. Kon AA, Shepard EK, Sederstrom NO et al. Defining futile and potentially inappropriate interventions: a policy statement from the Society of Critical Care Medicine Ethics Committee. *Crit Care Med* 2016;44:1769-1774.
6. Gross J, Williams B, Fade P, Brett SJ. Intensive care: balancing risk and benefit to facilitate informed decisions. *BMJ* 2018;363:k4135.
7. Escher M, Cullati S, Nendaz M, et al. Admission to intensive care: a qualitative study of triage and its determinants. *Health Serv Res* 2019;54:474-483.
8. Hsieh HF, Shannon SE. Three approaches to qualitative content analysis. *Qual Health Res* 2005;15:1277-1288.

For peer review only - http://bmjopen.bmj.com/site/about/guidelines.xhtml

9. Rubin EB, Buehler A, Halpern SD. Seriously ill patients' willingness to trade survival time to avoid high treatment intensity at the end of life. *JAMA Intern Med*, https://doi.org/10.1001/jamainternmed.2020.0681

10. Bassford CR, Krucien N, Ryan M et al. U.K. Intensivists' preferences for patient admission to ICU: evidence from a choice experiment. *Critical Care Med* 2019;47:1522-1530.

11. Escher M, Perneger TV, Heidegger CP, et al. Admission of incompetent patients to intensive care: doctors' responsiveness to family wishes. *Crit Care Med* 2009;37:528-532.

12. Zaros MC, Curtis JR, Silveira MJ, et al. Opportunity lost: end-of-life discussions in cancer patients who die in the hospital. *J Hosp Med* 2013;8:334-340.

13. Fritsch J, Petronio S, Helft PR, et al. Making decisions for hospitalized older adults: ethical factors considered by family surrogates. *J Clin Ethics* 2013;24:125-134.

14. Vink EE, Azoulay E, Caplan A et al. Time-limited trial of intensive care treatment: an overview of current literature. *Intensive Care Med* 2018;44:1369-1377.

For peer review only

Figure. Triage to intensive care: clinical scenarios and determinants of the decision in case of potentially nonbeneficial treatment

Legend

[ ] : determinant

→ : determines

– : composed of

? : is it concordant?

=

TI: treatment intensity deemed appropriate

For peer review only

For peer review only - http://bmjopen.bmj.com/site/about/guidelines.xhtml

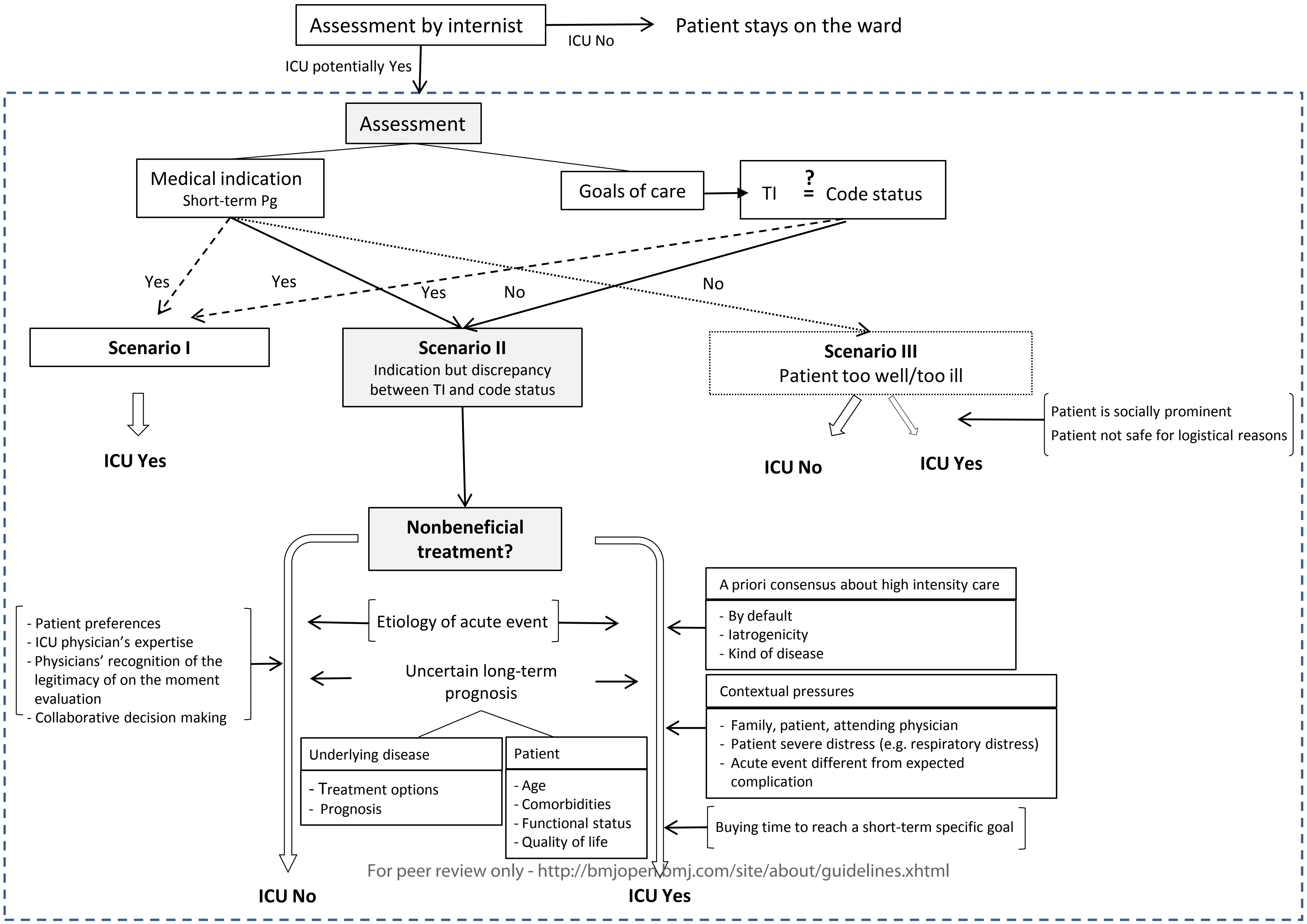

For peer review only - http://bmjopen.bmj.com/site/about/guidelines.xhtml

Supplementary file: Interview guide

Please tell me about the first / second situation you have chosen to discuss today.

Prompts:

- Patient characteristics: age? underlying illness? goals of care?
- Context: when dit it occur? what was the reason for calling the ICU? was the patient admitted to the ICU?
- Interactions between the internal medicine and the intensive care physicians:
    - Did the ICU physician come to see the patient?
    - Did you know the other physician?
    - Was the other physician senior or junior to you?
    - What were your expectations with respect to the other physician?

In your opinion, what made the decision-making process easier or more difficult in this situation?

Could you compare the two situations? (after the participant had discussed the two situations)

In your opinion, what is an ideal ICU admission decision-making process?

For peer review only

For peer review only - http://bmjopen.bmj.com/site/about/guidelines.xhtml

International Journal for Quality in Health Care; **Volume 19, Number 6**: pp. 349–357 10.1093/intqhc/mzm042
Advance Access Publication: 14 September 2007

Consolidated criteria for reporting qualitative research (COREQ): a 32-item checklist for interviews and focus groups

ALLISON TONG[1,2], PETER SAINSBURY[1,3] AND JONATHAN CRAIG[1,2]

[1]School of Public Health, University of Sydney, NSW 2006, Australia, [2]Centre for Kidney Research, The Children's Hospital at Westmead, NSW 2145, Australia, and [3]Population Health, Sydney South West Area Health Service, NSW 2170, Australia

Abstract

Background. Qualitative research explores complex phenomena encountered by clinicians, health care providers, policy makers and consumers. Although partial checklists are available, no consolidated reporting framework exists for any type of qualitative design.

Objective. To develop a checklist for explicit and comprehensive reporting of qualitative studies (indepth interviews and focus groups).

Methods. We performed a comprehensive search in Cochrane and Campbell Protocols, Medline, CINAHL, systematic reviews of qualitative studies, author or reviewer guidelines of major medical journals and reference lists of relevant publications for existing checklists used to assess qualitative studies. Seventy-six items from 22 checklists were compiled into a comprehensive list. All items were grouped into three domains: (i) research team and reflexivity, (ii) study design and (iii) data analysis and reporting. Duplicate items and those that were ambiguous, too broadly defined and impractical to assess were removed.

Results. Items most frequently included in the checklists related to sampling method, setting for data collection, method of data collection, respondent validation of findings, method of recording data, description of the derivation of themes and inclusion of supporting quotations. We grouped all items into three domains: (i) research team and reflexivity, (ii) study design and (iii) data analysis and reporting.

Conclusions. The criteria included in COREQ, a 32-item checklist, can help researchers to report important aspects of the research team, study methods, context of the study, findings, analysis and interpretations.

Keywords: focus groups, interviews, qualitative research, research design

For peer review only

Qualitative research explores complex phenomena encountered by clinicians, health care providers, policy makers and consumers in health care. Poorly designed studies and inadequate reporting can lead to inappropriate application of qualitative research in decision-making, health care, health policy and future research.

Formal reporting guidelines have been developed for randomized controlled trials (CONSORT) [1], diagnostic test studies (STARD), meta-analysis of RCTs (QUOROM) [2], observational studies (STROBE) [3] and meta-analyses of observational studies (MOOSE) [4]. These aim to improve the quality of reporting these study types and allow readers to better understand the design, conduct, analysis and findings of published studies. This process allows users of published research to be more fuller informed when they critically appraise studies relevant to each checklist and decide upon applicability of research findings to their local settings. Empiric studies have shown that the use of the CONSORT statement is associated with improvements in the quality of reports of randomized controlled trials [5]. Systematic reviews of qualitative research almost always show that key aspects of study design are not reported, and so there is a clear need for a CONSORT-equivalent for qualitative research [6].

The Uniform Requirements for Manuscripts Submitted to Biomedical Journals published by the International Committee of Medical Journal Editors (ICMJE) do not provide reporting guidelines for qualitative studies. Of all the mainstream biomedical journals (Fig. 1), only the British Medical Journal (BMJ) has criteria for reviewing qualitative research. However, the guidelines for authors specifically record that the checklist is not routinely used. In addition, the checklist is not comprehensive and does not provide specific guidance to assess some of the criteria. Although checklists for critical appraisal are available for qualitative research, there is no widely endorsed reporting framework for any type of qualitative research [7].

We have developed a formal reporting checklist for in-depth interviews and focus groups, the most common methods for data collection in qualitative health research.

Address reprint requests to: Allison Tong, Centre for Kidney Research, The Children's Hospital at Westmead, NSW 2145, Australia. Tel: +61-2-9845-1482; Fax: +61-2-9845-1491; E-mail: allisont@health.usyd.edu.au, allisont@chw.edu.au

© The Author 2007. Published by Oxford University Press on behalf of International Society for Quality in Health Care; all rights reserved

For peer review only - http://bmjopen.bmj.com/site/about/guidelines.xhtml

Downloaded from https://academic.oup.com/intqhc/article-abstract/19/6/349/1791966 by guest on 16 May 2020

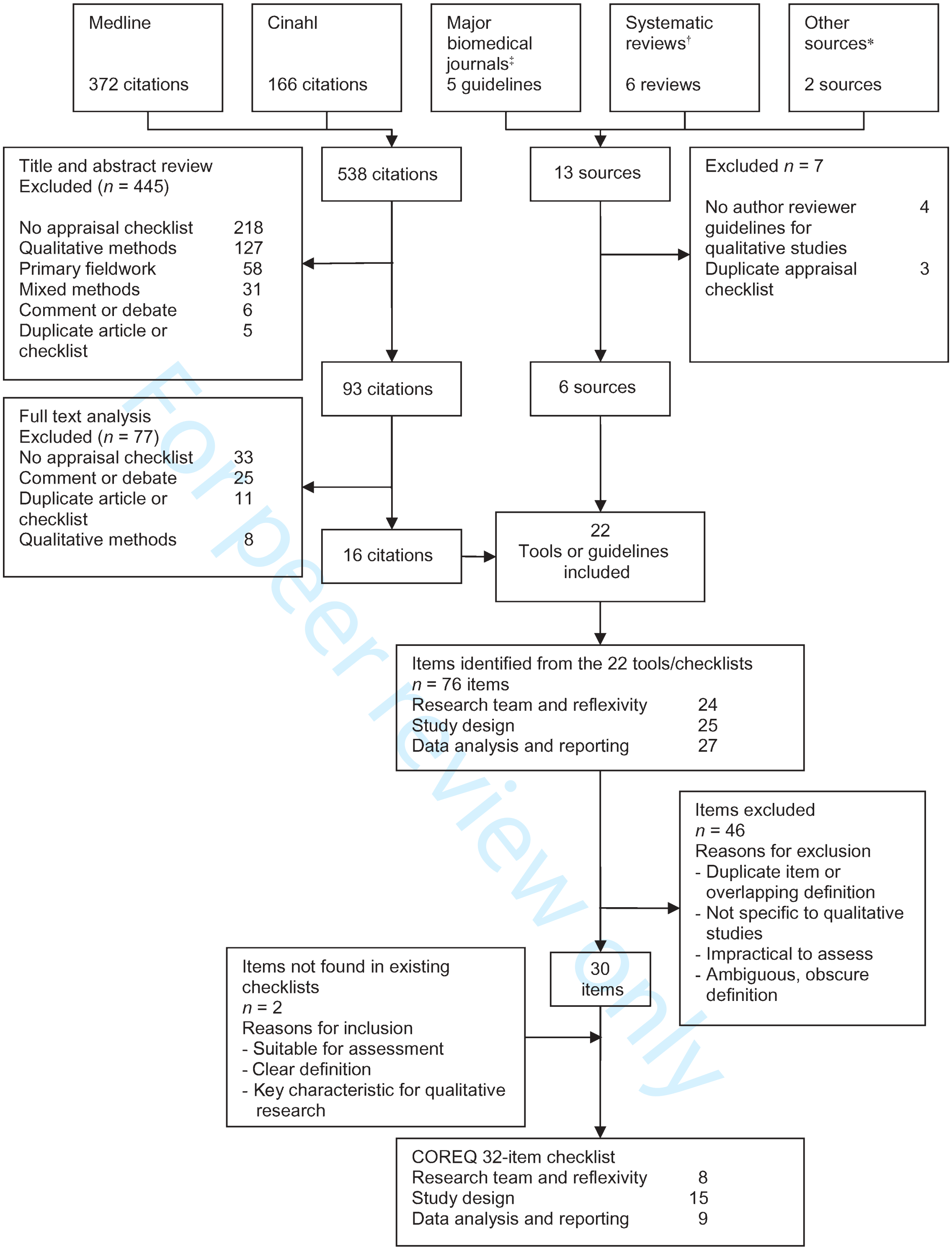

Figure 1 Development of the COREQ Checklist. *References [26, 27], †References [6, 28–32], ‡Author and reviewer guidelines provided by BMJ, JAMA, Lancet, Annals of Internal Medicine, NEJM.

These two methods are particularly useful for eliciting patient and consumer priorities and needs to improve the quality of health care [8]. The checklist aims to promote complete and transparent reporting among researchers and indirectly improve the rigor, comprehensiveness and credibility of interview and focus-group studies.

Basic definitions

Qualitative studies use non-quantitative methods to contribute new knowledge and to provide new perspectives in health care. Although qualitative research encompasses a broad range of study methods, most qualitative research

Downloaded from https://academic.oup.com/intqhc/article-abstract/19/6/349/1791966 by guest on 16 May 2020
For peer review only

For peer review only - http://bmjopen.bmj.com/site/about/guidelines.xhtml

publications in health care describe the use of interviews and focus groups [8].

Interviews

In-depth and semi-structured interviews explore the experiences of participants and the meanings they attribute to them. Researchers encourage participants to talk about issues pertinent to the research question by asking open-ended questions, usually in one-to-one interviews. The interviewer might re-word, re-order or clarify the questions to further investigate topics introduced by the respondent. In qualitative health research, in-depth interviews are often used to study the experiences and meanings of disease, and to explore personal and sensitive themes. They can also help to identify potentially modifiable factors for improving health care [9].

Focus groups

Focus groups are semi-structured discussions with groups of 4–12 people that aim to explore a specific set of issues [10]. Moderators often commence the focus group by asking broad questions about the topic of interest, before asking the focal questions. Although participants individually answer the facilitator's questions, they are encouraged to talk and interact with each other [11]. This technique is built on the notion that the group interaction encourages respondents to explore and clarify individual and shared perspectives [12]. Focus groups are used to explore views on health issues, programs, interventions and research.

Methods

Development of a checklist

Search strategy. We performed a comprehensive search for published checklists used to assess or review qualitative studies, and guidelines for reporting qualitative studies in: Medline (1966—Week 1 April 2006), CINAHL (1982—Week 3 April 2006), Cochrane and Campbell protocols, systematic reviews of qualitative studies, author or reviewer guidelines of major medical journals and reference lists of relevant publications. We identified the terms used to index the relevant articles already in our possession and performed a broad search using those search terms. The electronic databases were searched using terms and text words for research (standards), health services research (standards) and qualitative studies (evaluation). Duplicate checklists and detailed instructions for conducting and analysing qualitative studies were excluded.

Data extraction. From each of the included publications, we extracted all criteria for assessing or reporting qualitative studies. Seventy-six items from 22 checklists were compiled into a comprehensive list. We recorded the frequency of each item across all the publications. Items most frequently included in the checklists related to sampling method, setting for data collection, method of data collection, respondent validation of findings, method of recording data, description of the derivation of themes and inclusion of supporting quotations. We grouped all items into three domains: (i) research team and reflexivity, (ii) study design and (iii) data analysis and reporting. (see Tables 2–4)

Within each domain we simplified all relevant items by removing duplicates and those that were ambiguous, too broadly defined, not specific to qualitative research, or impractical to assess. Where necessary, the remaining items were rephrased for clarity. Based upon consensus among the authors, two new items that were considered relevant for reporting qualitative research were added. The two new items were identifying the authors who conducted the interview or focus group and reporting the presence of non-participants during the interview or focus group. The COREQ checklist for explicit and comprehensive reporting of qualitative studies consists of 32 criteria, with a descriptor to supplement each item (Table 1).

COREQ: content and rationale (see Tables 1)

Domain 1: research team and reflexivity

(i) Personal characteristics: Qualitative researchers closely engage with the research process and participants and are therefore unable to completely avoid personal bias. Instead researchers should recognize and clarify for readers their identity, credentials, occupation, gender, experience and training. Subsequently this improves the credibility of the findings by giving readers the ability to assess how these factors might have influenced the researchers' observations and interpretations [13–15].

(ii) Relationship with participants: The relationship and extent of interaction between the researcher and their participants should be described as it can have an effect on the participants' responses and also on the researchers' understanding of the phenomena [16]. For example, a clinician–researcher may have a deep understanding of patients' issues but their involvement in patient care may inhibit frank discussion with patient–participants when patients believe that their responses will affect their treatment. For transparency, the investigator should identify and state their assumptions and personal interests in the research topic.

Domain 2: study design

(i) Theoretical framework: Researchers should clarify the theoretical frameworks underpinning their study so readers can understand how the researchers explored their research questions and aims. Theoretical frameworks in qualitative research include: grounded theory, to build theories from the data; ethnography, to understand the culture of groups with shared characteristics; phenomenology, to describe the meaning and significance of experiences; discourse analysis, to analyse linguistic expression; and content analysis, to systematically organize data into a structured format [10].

Downloaded from https://academic.oup.com/intqhc/article-abstract/19/6/349/1791966 by guest on 16 May 2020

For peer review only

Table 1 Consolidated criteria for reporting qualitative studies (COREQ): 32-item checklist

No	Item	Guide questions/description
**Domain 1: Research team and reflexivity**		
Personal Characteristics		
1.	Interviewer/facilitator	Which author/s conducted the interview or focus group?
2.	Credentials	What were the researcher's credentials? *E.g. PhD, MD*
3.	Occupation	What was their occupation at the time of the study?
4.	Gender	Was the researcher male or female?
5.	Experience and training	What experience or training did the researcher have?
Relationship with participants		
6.	Relationship established	Was a relationship established prior to study commencement?
7.	Participant knowledge of the interviewer	What did the participants know about the researcher? e.*g. personal goals, reasons for doing the research*
8.	Interviewer characteristics	What characteristics were reported about the interviewer/facilitator? e.g. *Bias, assumptions, reasons and interests in the research topic*
**Domain 2: study design**		
Theoretical framework		
9.	Methodological orientation and Theory	What methodological orientation was stated to underpin the study? *e.g. grounded theory, discourse analysis, ethnography, phenomenology, content analysis*
Participant selection		
10.	Sampling	How were participants selected? *e.g. purposive, convenience, consecutive, snowball*
11.	Method of approach	How were participants approached? e.*g. face-to-face, telephone, mail, email*
12.	Sample size	How many participants were in the study?
13.	Non-participation	How many people refused to participate or dropped out? Reasons?
Setting		
14.	Setting of data collection	Where was the data collected? e.*g. home, clinic, workplace*
15.	Presence of non-participants	Was anyone else present besides the participants and researchers?
16.	Description of sample	What are the important characteristics of the sample? *e.g. demographic data, date*
Data collection		
17.	Interview guide	Were questions, prompts, guides provided by the authors? Was it pilot tested?
18.	Repeat interviews	Were repeat interviews carried out? If yes, how many?
19.	Audio/visual recording	Did the research use audio or visual recording to collect the data?
20.	Field notes	Were field notes made during and/or after the interview or focus group?
21.	Duration	What was the duration of the interviews or focus group?
22.	Data saturation	Was data saturation discussed?
23.	Transcripts returned	Were transcripts returned to participants for comment and/or correction?
**Domain 3: analysis and findings**z		
Data analysis		
24.	Number of data coders	How many data coders coded the data?
25.	Description of the coding tree	Did authors provide a description of the coding tree?
26.	Derivation of themes	Were themes identified in advance or derived from the data?
27.	Software	What software, if applicable, was used to manage the data?
28.	Participant checking	Did participants provide feedback on the findings?
Reporting		
29.	Quotations presented	Were participant quotations presented to illustrate the themes / findings? Was each quotation identified? e.*g. participant number*
30.	Data and findings consistent	Was there consistency between the data presented and the findings?
31.	Clarity of major themes	Were major themes clearly presented in the findings?
32.	Clarity of minor themes	Is there a description of diverse cases or discussion of minor themes?

(ii) Participant selection: Researchers should report how participants were selected. Usually purposive sampling is used which involves selecting participants who share particular characteristics and have the potential to provide rich, relevant and diverse data pertinent to the research question [13, 17]. Convenience sampling is less optimal because it may fail to capture important perspectives from difficult-to-reach people [16]. Rigorous attempts to recruit participants and reasons for non-participation should be stated to reduce the likelihood of making unsupported statements [18].

For peer review only

Downloaded from https://academic.oup.com/intqhc/article-abstract/19/6/349/1791966 by guest on 16 May 2020

For peer review only - http://bmjopen.bmj.com/site/about/guidelines.xhtml

Table 2 Items included in 22 published checklists: Research team and reflexivity domain

Item	References																					
	[26][a]	[27][a]	[6][b]	[28][b]	[32][b]	[13]	[15]	[14]	[17]	[33]	[34]	[35]	[16]	[19]	[36]	[7]	[37]	[23]	[38]	[39]	[22]	BMJ
Research team and reflexivity																						
Nature of relationship between the researcher and participants		•		•	•		•		•						•				•			
Examination of role, bias, influence	•	•			•	•	•	•							•						•	
Description of role		•		•					•	•				•	•					•	•	
Identity of the interviewer		•		•		•					•		•		•							
Continued and prolonged engagement		•				•							•	•					•	•		
Response to events	•	•				•	•	•														
Prior assumptions and experience		•						•									•			•		
Professional status		•					•								•							
Journal, record of personal experience		•								•				•								
Effects of research on researcher		•				•	•															
Qualifications		•													•							
Training of the interviewer/facilitator			•		•																	
Expertise demonstrated		•																	•			
Perception of research at inception								•						•								
Age							•															
Gender							•															
Social class							•															
Reasons for conducting study		•																				
Sufficient contact													•									
Too close to participants													•									
Empathy																	•					
Distance between researcher and participants							•															
Background								•														
Familiarity with setting																					•	

[a]Other publications, [b]Systematic review of qualitative studies; BMJ, British Medical Journal—editor's checklist for appraising qualitative research); •, item included in the checklist.

Downloaded from https://academic.oup.com/intqhc/article-abstract/19/6/349/1791966 by guest on 16 May 2020

Downloaded from https://academic.oup.com/intqhc/article-abstract/19/6/349/1791966 by guest on 16 May 2020

Table 3 Items included in 22 published checklists: Study design

Item	References																					
	[26][a]	[27][a]	[6][b]	[28][b]	[32][b]	[13]	[15]	[14]	[17]	[33]	[34]	[35]	[16]	[19]	[36]	[7]	[37]	[23]	[38]	[39]	[22]	BMJ
Study design																						
Methodological orientation, ontological or epistemological basis		•		•				•	•					•					•	•	•	•
Sampling—convenience, purposive	•	•			•	•	•	•	•	•	•	•	•	•		•		•	•	•	•	•
Setting		•		•	•			•			•				•					•		
Characteristics and description of sample		•			•			•			•		•	•								
Reasons for participant selection	•	•				•		•			•											
Non-participation	•	•		•	•																	
Inclusion and exclusion, criteria		•			•	•													•			
Identity of the person responsible for recruitment				•	•						•				•							
Sample size		•		•	•						•										•	
Method of approach		•									•					•						
Description of explanation of research to participants	•				•										•							
Level and type of participation														•								
Method of data collection, e.g. focus group, in-depth interview	•	•	•	•	•	•		•	•		•	•	•	•		•				•	•	
Audio and visual recording	•	•	•	•	•	•				•	•		•						•		•	•
Transcripts			•	•	•	•			•		•		•						•			•
Setting and location	•	•		•	•		•		•		•				•						•	•
Saturation of data	•	•	•			•			•				•	•							•	
Use of a topic guide, tools, questions	•	•	•								•					•			•	•		
Field notes			•	•	•	•													•			•
Changes and modifications	•	•		•	•														•		•	
Duration of interview, focus group		•				•					•									•		
Sensitive to participant language and views		•										•		•								
Number of interviews, focus groups		•				•																
Time span																					•	
Time and resources available to the study		•																				

[a]Other publications, [b]Systematic review of qualitative studies; BMJ, British Medical Journal—editor's checklist for appraising qualitative research; •, item included in the checklist.

Table 4 Items included in 22 published checklists: Analysis and reporting

Item	References																					
	[26][a]	[27][a]	[6][b]	[28][b]	[32][b]	[13]	[15]	[14]	[17]	[33]	[34]	[35]	[16]	[19]	[36]	[7]	[37]	[23]	[38]	[39]	[22]	BMJ
Respondent validation	•	•	•		•		•		•	•			•	•			•	•	•	•		
Limitations and generalizability	•	•		•	•		•		•		•		•	•				•	•			
Triangulation	•	•		•	•	•	•	•	•					•			•		•			
Original data, quotation		•	•	•	•			•	•		•			•		•				•	•	•
Derivation of themes explicit	•	•	•	•	•		•	•			•								•			•
Contradictory, diverse, negative cases	•	•		•	•		•			•				•					•			•
Number of data analysts	•	•	•			•			•			•	•						•			•
In-depth description of analysis	•			•	•			•			•			•							•	•
Sufficient supporting data presented	•	•		•	•		•				•					•						
Data, interpretation and conclusions linked and integrated		•		•	•							•		•						•		
Retain context of data		•					•	•						•					•			
Explicit findings, presented clearly	•	•		•					•	•												
Outside checks													•	•				•	•			
Software used		•				•													•			•
Discussion both for and against the researchers' arguments	•	•		•	•																	
Development of theories, explanations		•					•			•		•										
Numerical data		•									•							•				•
Coding tree or coding system		•					•												•		•	
Inter-observer reliability		•									•										•	
Sufficient insight into meaning/perceptions of participants		•								•												
Reasons for selection of data to support findings		•			•																	
New insight		•						•														
Results interpreted in credible, innovative way									•													
Eliminate other theories													•									
Range of views														•								
Distinguish between researcher and participant voices								•														
Proportion of data taken into account														•								

[a]Other publications, [b]Systematic review of qualitative studies; BMJ, British Medical Journal—editor's checklist for appraising qualitative research, •, item included in the checklist.

Downloaded from https://academic.oup.com/intqhc/article-abstract/19/6/349/1791966 by guest on 16 May 2020

For peer review only

Researchers should report the sample size of their study to enable readers to assess the diversity of perspectives included.

(iii) Setting: Researchers should describe the context in which the data were collected because it illuminates why participants responded in a particular way. For instance, participants might be more reserved and feel disempowered talking in a hospital setting. The presence of non-participants during interviews or focus groups should be reported as this can also affect the opinions expressed by participants. For example, parent interviewees might be reluctant to talk on sensitive topics if their children are present. Participant characteristics, such as basic demographic data, should be reported so readers can consider the relevance of the findings and interpretations to their own situation. This also allows readers to assess whether perspectives from different groups were explored and compared, such as patients and health care providers [13, 19].

(iv) Data collection: The questions and prompts used in data collection should be provided to enhance the readers' understanding of the researcher's focus and to give readers the ability to assess whether participants were encouraged to openly convey their viewpoints. Researchers should also report whether repeat interviews were conducted as this can influence the rapport developed between the researcher and participants and affect the richness of data obtained. The method of recording the participants' words should be reported. Generally, audio recording and transcription more accurately reflect the participants' views than contemporaneous researcher notes, more so if participants checked their own transcript for accuracy [19–21]. Reasons for not audio recording should be provided. In addition, field notes maintain contextual details and non-verbal expressions for data analysis and interpretation [19, 22]. Duration of the interview or focus group should be reported as this affects the amount of data obtained. Researchers should also clarify whether participants were recruited until no new relevant knowledge was being obtained from new participants (data saturation) [23, 24].

Domain 3: analysis and findings

(i) Data analysis: Specifying the use of multiple coders or other methods of researcher triangulation can indicate a broader and more complex understanding of the phenomenon. The credibility of the findings can be assessed if the process of coding (selecting significant sections from participant statements), and the derivation and identification of themes are made explicit. Descriptions of coding and memoing demonstrate how the researchers perceived, examined and developed their understanding of the data [17, 19]. Researchers sometimes use software packages to assist with storage, searching and coding of qualitative data. In addition, obtaining feedback from participants on the research findings adds validity to the researcher's interpretations by ensuring that the participants' own meanings and perspectives are represented and not curtailed by the researchers' own agenda and knowledge [23].

(ii) Reporting: If supporting quotations are provided, researchers should include quotations from different participants to add transparency and trustworthiness to their findings and interpretations of the data [17]. Readers should be able to assess the consistency between the data presented and the study findings, including the both major and minor themes. Summary findings, interpretations and theories generated should be clearly presented in qualitative research publications.

Discussion

The COREQ checklist was developed to promote explicit and comprehensive reporting of qualitative studies (interviews and focus groups). The checklist consists of items specific to reporting qualitative studies and precludes generic criteria that are applicable to all types of research reports. COREQ is a comprehensive checklist that covers necessary components of study design, which should be reported. The criteria included in the checklist can help researchers to report important aspects of the research team, study methods, context of the study, findings, analysis and interpretations.

At present, we acknowledge there is no empiric basis that shows that the introduction of COREQ will improve the quality of reporting of qualitative research. However this is no different than when CONSORT, QUOROM and other reporting checklists were introduced. Subsequent research has shown that these checklists have improved the quality of reporting of study types relevant to each checklist [5, 25], and we believe that the effect of COREQ is likely to be similar. Despite differences in the objectives and methods of quantitative and qualitative methods, the underlying aim of transparency in research methods and, at the least, the theoretical possibility of the reader being able to duplicate the study methods should be the aims of both methodological approaches. There is a perception among research funding agencies, clinicians and policy makers, that qualitative research is 'second class' research. Initiatives like COREQ are designed to encourage improvement in the quality of reporting of qualitative studies, which will indirectly lead to improved conduct, and greater recognition of qualitative research as inherently equal scientific endeavor compared with quantitative research that is used to assess the quality and safety of health care. We invite readers to comment on COREQ to improve the checklist.

References

1. Moher D, Schulz KF, Altman D. The CONSORT statement: revised recommendations for improving the quality of reports of parallel-group randomized trials. *JAMA* 2001;**285**:1987–91.

2. Moher D, Cook DJ, Eastwood S *et al.* Improving the quality of reports of meta-analyses of randomised controlled trials: the QUOROM statement. Quality of Reporting of Meta-analyses. *Lancet* 1999;**354**:1896–900.

Downloaded from https://academic.oup.com/intqhc/article-abstract/19/6/349/1791966 by guest on 16 May 2020

For peer review only

3. STROBE Statement: Strengthening the reporting of observational studies in epidemiology. http://www.strobe-statement.org/Checkliste.html

4. Stroup DF, Berlin JA, Morton SC *et al*. Meta-analysis of observational studies in epidemiology: a proposal for reporting. Meta-analysis Of Observational Studies in Epidemiology (MOOSE) group. *JAMA* 2000;**283**:2008–12.

5. Moher D, Jones A, Lepage L. Use of the CONSORT Statement and quality of reports of randomized trials. A comparative before-and-after evaluation. *JAMA* 2001;**285**:1992–5.

6. Mills E, Jadad AR, Ross C *et al*. Systematic review of qualitative studies exploring parental beliefs and attitudes toward childhood vaccination identified common barriers to vaccination. *J Clin Epidemiol* 2005;**58**:1081–8.

7. Knafl KA, Howard NJ. Interpreting and reporting qualitative research. *Res Nurs Health* 1984;**7**:7–14.

8. Sofaer S. Qualitative research methods. *Int J Qual Health Care* 2002;**14**:329–36.

9. Wright EB, Holcombe C, Salmon P. Doctor's communication of trust, care, and respect in breast cancer: qualitative study. *BMJ* 2004;**328**:864–8.

10. Liamputtong P, Ezzy D. *Qualitative Research Methods*. 2nd edn. Melbourne, Victoria: Oxford University Press, 2005.

11. Krueger RA, Casey MA. *Focus Groups. A Practical Guide for Applied Research*. Thousand Oaks CA: Sage Publications, 2000.

12. Morgan DL. *Focus Groups as Qualitative Research*. Newbury Park, California: Sage, 1988.

13. Giacomini MK, Cook DJ. Users' guides to the medical literature XXIII. Qualitative research in health care. A. Are the results of the study valid? *JAMA* 2000;**284**:357–62.

14. Malterud K. Qualitative research:standards challenges guidelines. *Lancet* 2001;**358**:483–8.

15. Mays N, Pope C. Qualitative research in health care: assessing quality in qualitative research. *BMJ* 2000;**320**:50–2.

16. Elder NC, William L. Reading and evaluating qualitative research studies. *J Fam Pract* 1995;**41**:279–85.

17. Cote L, Turgeon J. Appraising qualitative research articles in medicine and medical education. *Med Teach* 2005;**27**:71–5.

18. Altheide D, Johnson J. Criteria for assessing interpretive validity in qualitative research. In Denzin N, Lincoln Y (eds). *Handbook of Qualitative Research*. Thousand Oaks CA: Sage Publications, 1994.

19. Fossey E, Harvey C, McDermott F *et al*. Understanding and evaluating qualitative research. *Aust N Z J Psychiatry* 2002;**36**:717–32.

20. Seale C, Silverman S. Ensuring rigour in qualitative research. *Eur J Public Health* 1997;**7**:379–84.

21. Scheff T. Single case analysis in the health sciences. *Eur J Public Health* 1995;**5**:72–4.

22. Bluff R. Evaluating qualitative research. *Br J Midwifery* 1997;**5**:232–5.

23. Popay J, Rogers A, Williams G. Rationale and standards for the systematic review of qualitative literature in health services research. *Qual Health Res* 1998;**8**:341–51.

24. Blumer H. *Critiques of Research, in the Social Sciences*. New Brunswick, NJ: Transaction Books, 1979.

25. Delaney A, Bagshaw SM, Ferland A *et al*. A systematic evaluation of the quality of meta-anlyses in the critical care literature. *Crit Care* 2005;**9**:575–82.

26. Critical Skills Appraisal Programme (CASP) 10 Questions to help you make sense of qualitative research: Milton Keynes Primary Care Trust, 2002.

27. Spencer L, Ritchie J, Lewis J *et al*. *Quality in Qualitative Evaluation: A Framework for Assessing Research Evidence*. London: Cabinet Office. Government Chief Social Researcher's Office, 2003.

28. Campbell R, Pound P, Pope C *et al*. Evaluating meta-ethnography: a synthesis of qualitative research on lay experience of diabetes and diabetes care. *Soc Sci Med* 2003;**56**:671–84.

29. Feder GS, Hutson M, Ramsay I *et al*. Women exposed to intimate partner violence: expectations and experiences when they encounter health care professionals: a meta-analysis of qualitative studies. *Arch Intern Med* 2006;**166**:22–37.

30. Pound P, Britten N, Morgan M *et al*. Resisting medicines: a synthesis of qualitative studies of medicine taking. *Soc Sci Med* 2005;**61**:133–55.

31. Smith LK, Pope C, Botha JL. Patients' help-seeking experiences and delay in cancer presentation: a qualitative synthesis. *Lancet* 2005;**366**:825–31.

32. Walter FM, Emery J, Braithwaite D *et al*. Lay understanding of familial risk of common chronic diseases: a systematic review and synthesis of qualitative research. *Ann Fam Med* 2004; **2**:583–94.

33. Inui TS, Frankel RM. Evaluating the quality of qualitative research: a proposal pro-term. *J Gen Intern Med* 1991;**6**:485–6.

34. Boulton M, Fitzpatrick R, Swinburn C. Qualitative research in health care: II A structured review and evaluation of studies. *J Eval Clin Pract* 1996;**2**:171–9.

35. Dixon-Woods M, Shaw RL, Agarwal S *et al*. The problem of appraising qualitative research. *Qual Saf Health Care* 2004;**13**:223–5.

36. Hoddinott P, Pill R. A review of recently published qualitative research in general practice. More methodological questions than answers? *Fam Pract* 1997;**14**:313–9.

37. Kuzel AJ, Engel JD, Addison RB *et al*. Desirable features of qualitative research. *Fam Pract Res J* 1994;**14**:369–78.

38. Treloar C, Champness S, Simpson PL *et al*. Critical appraisal checklist for qualitative research studies. *Indian J Pediatr* 2000;**67**:347–51.

39. Cesario S, Morin K, Santa-Donato A. Evaluating the level of evidence in qualitative research. *J Obstet Gynecol Neonatal Nurs* 2001;**31**:708–14.

Accepted for publication 7 July 2007

For peer review only

Downloaded from https://academic.oup.com/intqhc/article-abstract/19/6/349/1791966 by guest on 16 May 2020

Physicians' perspective on potentially nonbeneficial treatment when assessing patients with advanced disease for ICU admission: a qualitative study.

Journal:	*BMJ Open*
Manuscript ID	bmjopen-2020-046268.R1
Article Type:	Original research
Date Submitted by the Author:	11-Mar-2021
Complete List of Authors:	Escher, Monica; University Hospitals Geneva, Division of Palliative Medicine; University of Geneva Faculty of Medicine, Unit for Development and Research in Medical Education (UDREM)<br>Nendaz, Mathieu; University of Geneva Faculty of Medicine, Unit of Development and Research in Medical Education (UDREM); University Hospitals Geneva, Division of General Internal Medicine<br>Cullati, Stéphane; University Hospitals Geneva, Quality of Care Service; University of Fribourg Faculty of Science and Medicine, Population Health Laboratory<br>Hudelson, Patricia; University Hospitals Geneva, Department of Primary Care
**Primary Subject Heading**:	Intensive care
Secondary Subject Heading:	Ethics, Health services research, Palliative care, Qualitative research
Keywords:	INTENSIVE & CRITICAL CARE, MEDICAL ETHICS, PALLIATIVE CARE, QUALITATIVE RESEARCH, Quality in health care < HEALTH SERVICES ADMINISTRATION & MANAGEMENT

SCHOLARONE™
Manuscripts

I, the Submitting Author has the right to grant and does grant on behalf of all authors of the Work (as defined in the below author licence), an exclusive licence and/or a non-exclusive licence for contributions from authors who are: i) UK Crown employees; ii) where BMJ has agreed a CC-BY licence shall apply, and/or iii) in accordance with the terms applicable for US Federal Government officers or employees acting as part of their official duties; on a worldwide, perpetual, irrevocable, royalty-free basis to BMJ Publishing Group Ltd ("BMJ") its licensees and where the relevant Journal is co-owned by BMJ to the co-owners of the Journal, to publish the Work in this journal and any other BMJ products and to exploit all rights, as set out in our licence.

The Submitting Author accepts and understands that any supply made under these terms is made by BMJ to the Submitting Author unless you are acting as an employee on behalf of your employer or a postgraduate student of an affiliated institution which is paying any applicable article publishing charge ("APC") for Open Access articles. Where the Submitting Author wishes to make the Work available on an Open Access basis (and intends to pay the relevant APC), the terms of reuse of such Open Access shall be governed by a Creative Commons licence – details of these licences and which Creative Commons licence will apply to this Work are set out in our licence referred to above.

Other than as permitted in any relevant BMJ Author's Self Archiving Policies, I confirm this Work has not been accepted for publication elsewhere, is not being considered for publication elsewhere and does not duplicate material already published. I confirm all authors consent to publication of this Work and authorise the granting of this licence.

For peer review only

Physicians' perspective on potentially nonbeneficial treatment when assessing patients with advanced disease for ICU admission: a qualitative study.

Monica Escher,[1,2] Mathieu Nendaz,[2,3] Stéphane Cullati,[1,4,5] Patricia Hudelson.[6]

1. Division of Palliative Medicine, Geneva University Hospitals, Geneva, Switzerland

2. Unit of Development and Research in Medical Education, Faculty of Medicine, University of Geneva, Geneva, Switzerland

3. Division of General Internal Medicine, Geneva University Hospitals, Geneva, Switzerland

4. Quality of Care Service, Geneva University Hospitals, Geneva, Switzerland

5. Population Health Laboratory (#PopHealthLab), Faculty of Science and Medicine, University of Fribourg, Fribourg, Switzerland

6. Division of Primary Care Medicine, Geneva University Hospitals, Switzerland

Corresponding author:

Monica Escher

Division of Palliative Medicine

Geneva University Hospitals

Rue Gabrielle-Perret-Gentil 4

1211 Geneva - Switzerland

E-mail address: monica.escher@hcuge.ch

Telephone number: +41 22 372 29 23

For peer review only

Word count

Abstract: 234

Text: 2969

Figures: 2

References: 25

For peer review only

For peer review only - http://bmjopen.bmj.com/site/about/guidelines.xhtml

Abstract

Objective: The use of intensive care at the end of life can be high, leading to inappropriate healthcare utilization, and prolonged suffering for patients and families. The objective of the study was to determine which factors influence physicians' admission decisions in situations of potentially nonbeneficial intensive care.

Design: This is a secondary analysis of a qualitative study exploring the triage process. In-depth interviews were analysed using an inductive approach to thematic content analysis.

Setting: Data were collected in a Swiss tertiary care center between March and June 2013.

Participants: 12 ICU physicians and 12 internists routinely involved in ICU admission decisions.

Results: Physicians struggled to understand the request for intensive care for patients with advanced disease and full code status. Physicians considered patients' long-term vital and functional prognosis, but they also resorted to shortcuts, i.e. a priori consensus about reasons for admitting a patient. Family pressure and unexpected critical events were determinants of admission to the ICU. Patient preferences, ICU physician's expertise and collaborative decision making facilitated refusal. Physicians were willing to admit a patient with advanced disease for a limited amount of time to fulfill a personal need.

Conclusions: In situations of potentially nonbeneficial intensive care, the influence of shortcuts or context-related factors suggests that practice variations and inappropriate admission decisions are likely to occur. Institutional guidelines and timely goals of care discussions with patients with advanced disease and their families could contribute to ensuring appropriate levels of care.

For peer review only - http://bmjopen.bmj.com/site/about/guidelines.xhtml

Strengths and limitations of this study

- Participant sample was representative of physicians involved in ICU admission decisions in our institution.
- In-depth interviews were conducted by an experienced medical sociologist.
- Data analysis was done by a multidisciplinary research team including clinicians from the intensive care, internal medicine, and palliative care fields, a medical sociologist, and a medical anthropologist.
- The main limitation of this study is that it is a secondary analysis of interviews that did not specifically focus on the role of potentially nonbeneficial treatment in ICU admission decisions.

For peer review only

Keywords: intensive care, medical futility, decision-making, , end of life care

Funding

This work was supported by the Swiss National Science Foundation, National Research Program "End of Life" (NRP 67) grant number 139304.

Competing interests statement

The authors declare no potential conflicts of interest with respect to the research, authorship, and/or publication of this article.

Data availability statement

All data relevant to the study are included in the article or uploaded as supplementary information. No additional data are available.

Ethics approval

This study was approved by the Geneva Research Ethics Committee (12-042). The participants gave informed consent before taking part in the study.

For peer review only

For peer review only - http://bmjopen.bmj.com/site/about/guidelines.xhtml

INTRODUCTION

The use of intensive care in the last month of life can be high, especially for non cancer patients.[1] Providing nonbeneficial treatments to patients with advanced disease only prolongs suffering at the end of life. It is associated with family distress[2] and healthcare staff burnout[3]. Potentially nonbeneficial interventions is a concern for patients cared for in intensive care units (ICU).[4,5] In 2015 several prominent professional societies, among which the American Thoracic Society and the European Society for Intensive Care Medicine, published a joint statement about how to respond to patients' or families' requests for potentially inappropriate treatments.[6] The term « potentially inappropriate » was recommended over « futile » since it was acknowledged that a patient's values and preferences can legitimately lead him or his family to request life-prolonging interventions when physicians consider those treatments to be inappropriate. The requested medical intervention must have some chance to achieve the patient's goal, and in this case the physicians' justifications for not providing it are ethically based. Of note, the statement does not give guidance about how to determine how much chance justifies to administer the requested treatment, or to challenge its appropriateness. The definition of potentially inappropriate interventions was addressed in a subsequent statement of the Society of Critical Care Medicine.[5] The medical interventions should allow to achieve at least one of two goals: either the patient will be able to live outside the acute care setting, or he will recover sufficient neurologic function to perceive the treatment benefits. Physicians' clinical judgment however is central to the decision since they have to estimate survival and cognitive outcomes. Moreover, the guidance makes allowance for time-limited interventions that might promote a patient's goals of care.

For peer review only - http://bmjopen.bmj.com/site/about/guidelines.xhtml

The discussion about potentially nonbeneficial interventions has mainly focused on the administration of treatments to patients staying in the ICU. However it can be an issue during triage. For example, no consensus was found about limiting the admission to intensive care based on a patient's chances of survival, not even for a chance as low as 0.1% or less.[7] The lack of specific criteria for ICU admission decisions has been recently pointed out.[8] Whereas a decision supporting framework was developed to address the issue of limiting or not life-sustaining treatments in the ICU,[9] no such framework exists for triage. Yet, deciding whether to admit a patient to the ICU is often complex, and physicians mostly rely on their clinical judgment.[10]

As significant knowledge gaps remain about the provision of potentially nonbeneficial care, more studies on the topic have been called for.[11] Triage is an important area in this respect. Numerous patient-, and context-related factors were shown to influence the decision to admit or not a patient to the ICU, but data are lacking about how these various factors come into play within the decision making process.[12,13] When physicians assess a critically ill patient for admission to the ICU, they evaluate the medical indication – i.e. added benefit of intensive care in terms of short-term prognosis - on the one hand, and long-term survival, potential for functional and cognitive recovery, and patient preferences on the other hand. The latter factors are framed in terms of goals of care. Based on their assessment, physicians determine what they think is appropriate treatment intensity for the patient. A particularly difficult situation involves critically ill patients with advanced disease for whom physicians consider limiting treatment intensity, and who have a full code status.[14] Although physicians have no obligation to follow a patient's code status, they cannot disregard it lightly. It is a strong indicator of treatment intensity, intended to guide decisions in case of an unexpected critical event. Therefore, to go against code status, i.e. not to admit

For peer review only - http://bmjopen.bmj.com/site/about/guidelines.xhtml

a patient to the ICU, is a difficult decision to make. We aimed to determine which factors physicians consider when they are faced with the ethical issue of providing potentially nonbeneficial intensive care to a critically ill patient, and how these factors influence ICU admission decisions.

For peer review only - http://bmjopen.bmj.com/site/about/guidelines.xhtml

METHODS

This is a secondary analysis of a qualitative study exploring the triage process.[14] The study was conducted at a tertiary care hospital. It was approved by the Geneva Research Ethics Committee.

Participants and data collection

Physicians working in the Divisions of General Internal Medicine and of Intensive Care, and routinely assessing patients for intensive care were eligible. We included physicians from the two specialties because triage is a collaborative process in our institution. The internal medicine physician gives the ICU physician the relevant clinical information and goals of care of the critically ill patient. The ICU physician personally evaluates the patient and gives expert advice. The two physicians discuss whether or not to admit the patient to intensive care, but the ICU physician usually has the final say.

We used a combination of convenience and snowball sampling, and included equal numbers of internists (n=12) and ICU physicians (n=12). Study participants were representative of the physicians who make ICU admission decisions in our institution. Internists included both certified chief residents (n=8) and residents (n=4), since the latter are involved in admission decisions during night calls. ICU physicians were chief residents (n=7) and attendings (n=5). Participants were recruited between March and June 2013 after we presented the study at staff meetings and through email invitations. Interested physicians contacted one of the researchers (SC). At the end of the interview they were asked whether they knew of a colleague who might participate. All the identified physicians accepted to be included in the study. The participants gave written consent to participate in the study.

For peer review only - http://bmjopen.bmj.com/site/about/guidelines.xhtml

The interview guide was pre-tested with two internists and two ICU physicians (Supplementary file). A male PhD medical sociologist (SC) conducted face-to-face in-depth interviews.He was a member of the research team, had extensive experience in qualitative research and an interest in interprofessional collaboration and sociology of healthcare professions. He had neither previous nor hierarchical relationships to the interviewees. All interviews took place at the hospital, in a dedicated room outside the Division of General Internal Medicine and the Division of Intensive Care. SC introduced himself to participants as a sociologist collaborating on the research project.

Participants were invited to reflect on their experience of two ICU admission decisions involving a medical inpatient. They were asked to choose significant cases with regards to the way the decision was made. We indicated that the decision itself – admission or no admission – was not important, that the decision making process could have gone either smoothly or not, and that the clinical situations could be simple or complex. During the interviews the participants sometimes freely referred to other clinical situations, either to make their point or to expand on the idea they were developing. The main objective of the study was to identify the factors that facilitated or hindered admission decisions. Interviews lasted 57 minutes on average (min 26, max 94). They were recorded, transcribed *verbatim* and anonymised. No field notes were taken and each physician was interviewed only once. Participants were not asked to read and react to the transcripts of their interviews , but 3 ICU physicians and 2 internists were presented with the main results of the study and asked whether they reflected their experiences.[14]

Analysis

Interview transcripts were analysed using an inductive approach to thematic content analysis.[15] This approach enables to identify meaningful information regarding the research

For peer review only - http://bmjopen.bmj.com/site/about/guidelines.xhtml

question from the textual data, and to relate it to overarching themes. Themes are analysed and interpreted into a coherent descriptive model. Analysis aimed to identify factors that influenced participants' decision-making around ICU admission. In particular, we were interested in understanding participants' views regarding potentially nonbeneficial intensive care ("medical futility").

Four interviews (two with internists, two with ICU physicians) were first independently read and then discussed by members of the research team (ME, SC, MN, PH). Based on this first reading, a preliminary list of codes was developed, and independently applied by SC and ME to the same 4 interviews. Any coding discrepancies were resolved by consensus, and a third researcher (PH or MN) cross-checked the coded interviews. A finalized codelist was then applied by ME or SC to the remaining interviews, and then codings were cross-checked by two researchers (ME or SC, and PH or MN). Whenever new ideas appeared in the interviews, new codes were created and then applied to all interviews. Codes were clustered according to their content relatedness (e.g. "intensive care as default option"). Coding and analysis were conducted using Atlas.ti Scientific Software Development (Version 7.0.71).

Patient and public involvement

No patients were involved.

For peer review only - http://bmjopen.bmj.com/site/about/guidelines.xhtml

RESULTS

Participant characteristics

Among the 24 physicians, 17 were male. Mean age was 38 years (range 27-51) and mean number of years since graduation was 11.8 (SD 6.8). On average ICU physicians were older and more experienced than internists. Three internal medicine residents had never worked in an ICU. Participants' characteristics reflected medical staff's training background and working organization in our institution. Most ICU physicians train in a primary specialty before training in critical care medicine. Since only senior ICU physicians evaluate critically ill patients on the wards, the differences in age and experience between intensivist and internist participants were expected.

Clinical situations during triage

Physicians described two scenarios, when the decision to admit or refuse a patient to intensive care was straightforward (Figure 1). Either there was a medical indication, i.e. short-term benefit, and high intensity care was considered appropriate and was congruent with code status, then the patient was admitted; or there was no medical indication, and then the patient was refused.

In situations where there was no medical indication, physicians explained that sometimes context-related factors, i.e. social pressure due to a patient's prominence, and concerns about patient's safety on the ward, could lead to the patient being admitted.

> *"Probably the patient would not have had any benefit from intensive care, … but sometimes we must admit [a patient], precisely when there is some doubt, because we choose the safe side." (ICU12)*

Participants explicitly raised the issue of potentially nonbeneficial intensive care when they reported being faced with a dilemma. The dilemma arose from the discrepancy

For peer review only - http://bmjopen.bmj.com/site/about/guidelines.xhtml

between their assessment – low intensity care more appropriate – and the high intensity care required by a full code status. It usually concerned patients with advanced disease as these patients could benefit from life-sustaining interventions, but their long-term survival prognosis and their capacities for cognitive and functional recovery were limited. (scenario II). In these situations, physicians struggled to make sense of the request for treatment.

"When the patient has a cancer at a very advanced stage and still, it is decided to intubate him because he has a pulmonary infection, is an admission to intensive care really meaningful?" (ICU01)

Factors influencing ICU admission decision in the case of potentially nonbeneficial treatments

Participants described factors that oriented a decision towards admission, towards refusal, or that were used for either decision (Figure 2). There was consensus among respondents that intensive care should be provided as a default option in cases of great uncertainty, for patients needing intensive care as a consequence of an iatrogenic event, or for patients with onco-hematological diseases.

"When in doubt, we admit and we treat." (ICU10)

"When there are so-called iatrogenic complications, I feel I have a responsibility to treat the complication, … to make abstraction of the patient's general context and to use all available means to take care of it." (MED11)

In addition, respondents reported that they could be pressured into admitting a patient with advanced disease by the family or the referring physician. Factors related to the acute event could also prompt physicians to admit a patient for whom limited treatment intensity had previously been decided.

For peer review only - http://bmjopen.bmj.com/site/about/guidelines.xhtml

164 *"Some families demand everything, even though it is futile,… and they put an enormous pressure on the system." (ICU04)*

166 Physicians were also willing to provide life-sustaining treatments to a terminally ill patient for a limited amount of time in order to fulfill a personal need of the patient or family.

169 *"Even in a desperate situation, we can admit a patient to intensive care if we know there is something coming up; we wait for a relative who is on his way" (ICU11)*

171 Determinants of ICU refusal in the case of potentially nonbeneficial treatment involved not only consideration of patient preferences but were also influenced by professional interactions. The ICU physician's expertise carried weight; collaborative decision making between internists and ICU physicians facilitated refusal as did physicians' recognition that ad hoc evaluation was at times as valuable as code status.

176 *"We decided to go against the code status. But we did it together, we evaluated the patient, we discussed" (MED01)*

178 In such cases, and depending on the type of the acute event, the patient could be admitted or not. Physicians also took into account long term prognosis. They considered patient-related factors, i.e. age, comorbidities, functional status and quality of life, and disease-related factors, i.e. prognosis, and availability of disease-directed treatments

For peer review only - http://bmjopen.bmj.com/site/about/guidelines.xhtml

DISCUSSION

Physicians in our study explicitly integrated the provision of potentially nonbeneficial treatment into the decision making process of ICU admission when they were faced with a dilemma. The dilemma concerned patients with advanced disease who were full code, but for whom physicians considered low intensity care to be more appropriate. In these situations physicians took many factors into account, which reflects how complex the decision may be. They reasoned about patient's long-term prognosis, but they also resorted to shortcuts, i.e. a priori consensus about reasons for admitting a patient. Human factors influenced the decision towards admission: physicians felt pressure on the part of the family and as a consequence of unexpected critical events. More positively, physicians were willing to admit a patient if it could enable him to reach a meaningful short-term goal. Professional factors facilitated the decision towards refusal of intensive care: medical expertise, in particular the ICU physician's, and collaborative decision making.

Our findings show that the provision of potentially nonbeneficial treatments can be an issue for physicians during triage as it is in the ICU.[4] The determinants of ICU admission or refusal in these situations are on the whole similar to the ones reported in the current literature about the general process of decision making for ICU admissions.[12,13] Physicians consider longer-term survival and functional outcomes, and are influenced by patient preferences, and context-related factors.

The use of short-cuts in admission decisions contrasts with the process advocated to decide about limiting or not life-sustaining treatments in the ICU.[9] It reflects the time-pressured context of triage when repeated meetings with the family and among the healthcare professionals are hardly feasible and when prognostic uncertainty is high. To admit a patient in case of great uncertainty is consistent with professional guidelines that

For peer review only - http://bmjopen.bmj.com/site/about/guidelines.xhtml

deem overtriage to be more acceptable than undertriage.[11] Admission when in doubt is a behavior physicians reported previously.[16] Physicians' response to unexpected events could be ethically problematic and lead to potentially inappropriate admissions to intensive care. It is likely that patients' perspectives differ from physicians' in this respect as a recent study has shown that patients are willing to trade survival time to avoid end of life in an ICU.[17] Family opinion has been shown to significantly influence ICU admission decisions.[14,18,19] Family can either act as useful healthcare surrogates or make requests in response to their own needs.[20,21] Interestingly physicians referred to family only as putting pressure towards admission in situations of potentially nonbeneficial treatments. It epitomizes the difficulty of responding to such requests, which has prompted the issuance of guidance by professional societies.[6] Disagreement between medical team and family has been associated with perceived inappropriate care in the ICU,[22] and also with potentially inappropriate admissions to the ICU from hospital wards[23]. Similarly to our study, pressure from the referring physician to provide potentially nonbeneficial treatments has been reported in the ICU setting.[22]

ICU physicians' expertise and collaborative decision making are factors that can facilitate a decision not to admit a patient. Clinician experience was also found to have a significant influence on challenging ICU admission decisions in a qualitative study about triage in the emergency department.[24] Such decisions are difficult to make and physicians' willingness to admit patients to the ICU so that they or their family could fulfill a personal need is in keeping with current attitudes. Time-limited trial is an accepted strategy for patients with a poor prognosis when survival benefit with intensive care or patient preferences are unclear, or when patient and/or family need time to adapt.[25] Such an

For peer review only - http://bmjopen.bmj.com/site/about/guidelines.xhtml

approach is concordant with the intention to provide patient- and family-centered sensitive care.

Our study has limitations. It is a secondary analysis of interviews that did not specifically focus on the role of potentially nonbeneficial treatment in ICU admission decision making. Other issues might arise in a more in-depth study on this topic. In addition, the study was conducted in a context where internists and ICU physicians collaborate when deciding on ICU admission. Where this is not the case, physicians may be influenced by different factors. Nonetheless, data about triage and the provision of potentially nonbeneficial treatments are scarce and our study brings novel insights into physicians' decision making under these time-pressured circumstances. We were able to identify several patient- , physician-, and context-related factors, and we could determine in which direction these various factors influenced the ICU admission decision.

CONCLUSION

Physicians are concerned about providing potentially nonbeneficial intensive care treatment for critically ill patients with advanced disease in situations of uncertainty. The ICU admission decision is then complex and influenced by a variety of medical and contextual factors. The role that shortcuts or context-related factors may play raises concerns about potentially inappropriate admission to intensive care. Our results highlight the risk of practice variation in ICU admission decisions. Additional research should focus on how physicians weigh multiple contextual factors, and on how institutional guidelines and advance care planning with patients and families can help admission decisions and contribute to ensuring appropriate levels of care.

For peer review only - http://bmjopen.bmj.com/site/about/guidelines.xhtml

Author contributions

M.E. contributed to study concepts. M.E., M.N, S.C., P.H. contributed to study design. S.C. and M.E. collected the data. All the authors contributed to quality control of the data. All authors contributed to data analysis and interpretation. M.E. drafted the manuscript. All authors contributed to manuscript editing and review.

Acknowledgments

The authors thank the physicians who participated in the study. We also thank Prof Thomas Perneger for his invaluable contribution to study concepts and design, and Prof Bara Ricou for her contribution to the whole project about the determination of factors influencing ICU admission decisions.

For peer review only

For peer review only - http://bmjopen.bmj.com/site/about/guidelines.xhtml

REFERENCES

1. Teno JM, Gozalo P, Trivedi AN, et al. Site of Death, Place of Care, and Health Care Transitions Among US Medicare Beneficiaries, 2000-2015. *JAMA.* 2018;320(3):264-271.
2. Chen C, Michaels J, Meeker MA. Family outcomes and perceptions of end-of-life care in the intensive care unit: a mixed-methods review. *Journal of palliative care.* 2019:825859719874767.
3. Schwarzkopf D, Ruddel H, Thomas-Ruddel DO, et al. Perceived Nonbeneficial Treatment of Patients, Burnout, and Intention to Leave the Job Among ICU Nurses and Junior and Senior Physicians. *Crit Care Med.* 2017;45(3):e265-e273.
4. Piers RD, Azoulay E, Ricou B, et al. Perceptions of appropriateness of care among European and Israeli intensive care unit nurses and physicians. *JAMA.* 2011;306(24):2694-2703.
5. Kon AA, Shepard EK, Sederstrom NO, et al. Defining Futile and Potentially Inappropriate Interventions: A Policy Statement From the Society of Critical Care Medicine Ethics Committee. *Crit Care Med.* 2016;44(9):1769-1774.
6. Bosslet GT, Pope TM, Rubenfeld GD, et al. An Official ATS/AACN/ACCP/ESICM/SCCM Policy Statement: Responding to Requests for Potentially Inappropriate Treatments in Intensive Care Units. *Am J Respir Crit Care Med.* 2015;191(11):1318-1330.
7. Sprung CL, Danis M, Iapichino G, et al. Triage of intensive care patients: identifying agreement and controversy. *Intensive Care Med.* 2013;39(11):1916-1924.
8. Dahine J, Hebert PC, Ziegler D, Chenail N, Ferrari N, Hebert R. Practices in Triage and Transfer of Critically Ill Patients: A Qualitative Systematic Review of Selection Criteria. *Crit Care Med.* 2020;48(11):e1147-e1157.
9. Kerckhoffs MC, Senekal J, van Dijk D, et al. Framework to Support the Process of Decision-Making on Life-Sustaining Treatments in the ICU: Results of a Delphi Study. *Crit Care Med.* 2020;48(5):645-653.

For peer review only

For peer review only - http://bmjopen.bmj.com/site/about/guidelines.xhtml

10. Gross J, Williams B, Fade P, Brett SJ. Intensive care: balancing risk and benefit to facilitate informed decisions. *BMJ.* 2018;363:k4135.

11. Nates JL, Nunnally M, Kleinpell R, et al. ICU Admission, Discharge, and Triage Guidelines: A Framework to Enhance Clinical Operations, Development of Institutional Policies, and Further Research. *Crit Care Med.* 2016;44(8):1553-1602.

12. Gopalan PD, Pershad S. Decision-making in ICU - A systematic review of factors considered important by ICU clinician decision makers with regard to ICU triage decisions. *J Crit Care.* 2019;50:99-110.

13. James FR, Power N, Laha S. Decision-making in intensive care medicine - A review. *J Intensive Care Soc.* 2018;19(3):247-258.

14. Escher M CS, Nendaz M, Ricou B, Hudelson P, Perneger TV, Dayer P. Admission to intensive care: a qualitative study of triage and its determinants. *Health Serv Res.* 2019;54(2):474-483.

15. Hsieh HF, Shannon SE. Three approaches to qualitative content analysis. *Qual Health Res.* 2005;15(9):1277-1288.

16. Oerlemans AJ, van Sluisveld N, van Leeuwen ES, Wollersheim H, Dekkers WJ, Zegers M. Ethical problems in intensive care unit admission and discharge decisions: a qualitative study among physicians and nurses in the Netherlands. *BMC Med Ethics.* 2015;16:9.

17. Rubin EB, Buehler A, Halpern SD. Seriously Ill Patients' Willingness to Trade Survival Time to Avoid High Treatment Intensity at the End of Life. *JAMA Intern Med.* 2020;180(6):907-909.

18. Bassford CR, Krucien N, Ryan M, et al. U.K. Intensivists' Preferences for Patient Admission to ICU: Evidence From a Choice Experiment. *Crit Care Med.* 2019;47(11):1522-1530.

19. Escher M, Perneger TV, Heidegger CP, Chevrolet JC. Admission of incompetent patients to intensive care: doctors' responsiveness to family wishes. *Crit Care Med.* 2009;37(2):528-532.

20. Zaros MC, Curtis JR, Silveira MJ, Elmore JG. Opportunity lost: end-of-life discussions in cancer patients who die in the hospital. *J Hosp Med.* 2013;8(6):334-340.

For peer review only

For peer review only - http://bmjopen.bmj.com/site/about/guidelines.xhtml

21. Fritsch J, Petronio S, Helft PR, Torke AM. Making decisions for hospitalized older adults: ethical factors considered by family surrogates. *J Clin Ethics.* 2013;24(2):125-134.

22. Piers RD, Azoulay E, Ricou B, et al. Inappropriate care in European ICUs: confronting views from nurses and junior and senior physicians. *Chest.* 2014;146(2):267-275.

23. Carter HE, Lee XJ, Gallois C, et al. Factors associated with non-beneficial treatments in end of life hospital admissions: a multicentre retrospective cohort study in Australia. *BMJ open.* 2019;9(11):e030955.

24. Emerson P, Brooks D, Quasim T, Puxty A, Kinsella J, Lowe DJ. Factors influencing intensive care admission: a mixed methods study of EM and ICU. *Eur J Emerg Med.* 2017;24(1):29-35.

25. Vink EE, Azoulay E, Caplan A, Kompanje EJO, Bakker J. Time-limited trial of intensive care treatment: an overview of current literature. *Intensive Care Med.* 2018;44(9):1369-1377.

For peer review only

For peer review only - http://bmjopen.bmj.com/site/about/guidelines.xhtml

Figure 1. Triage to intensive care: decision making scenarios

Legend

[ ]→: determinant

⇨: decision

– : composed of

HIT: high intensity treatment

LIT: low intensity treatment

For peer review only

For peer review only - http://bmjopen.bmj.com/site/about/guidelines.xhtml

Figure 2. Scenario II: Factors influencing the decision towards admission to or refusal of intensive care

Legend

[ ]→: determinant

⇨: decision

– : composed of

For peer review only

For peer review only - http://bmjopen.bmj.com/site/about/guidelines.xhtml

Figure 1. Triage to intensive care: decision making scenarios

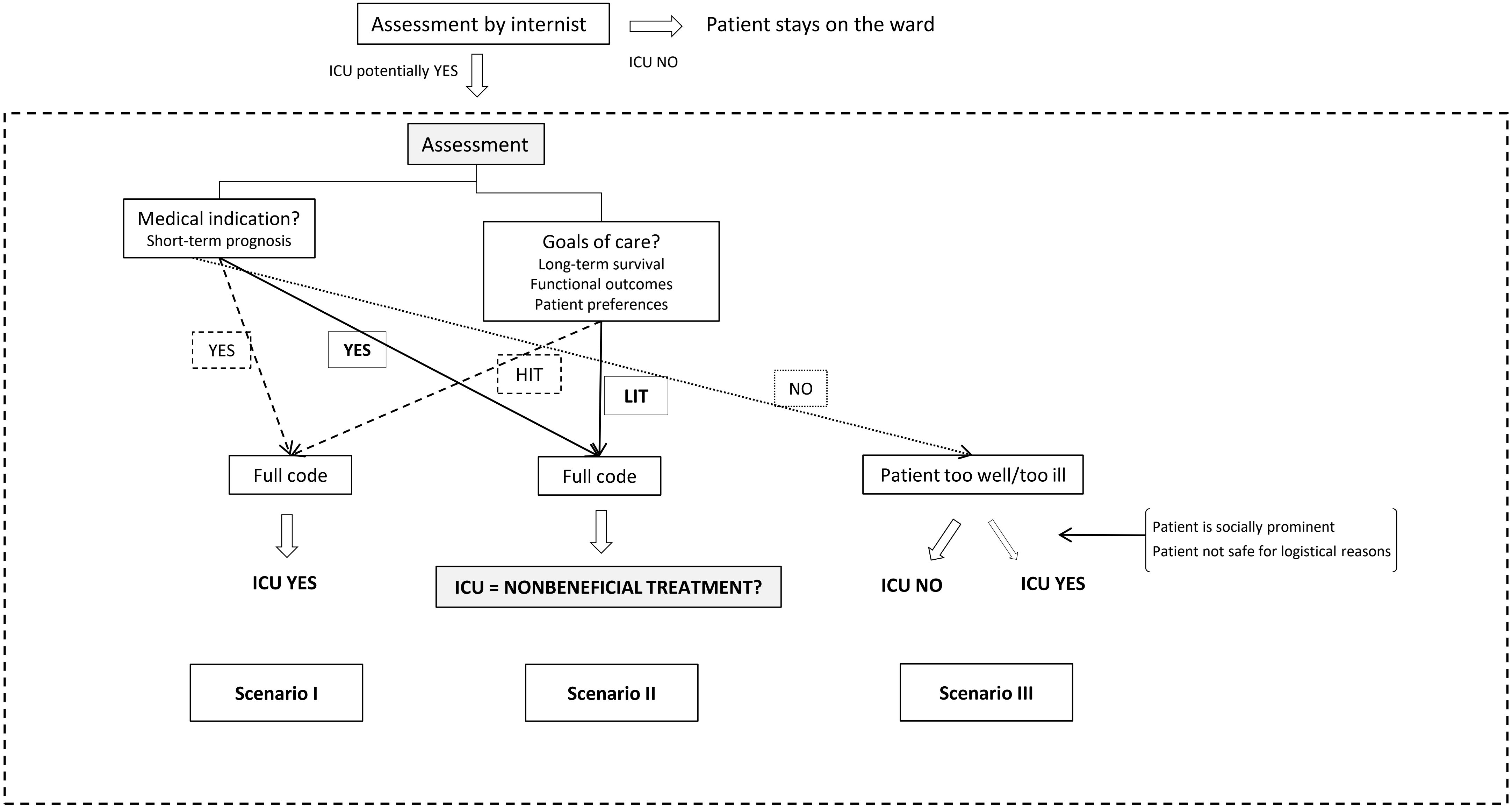

Figure 2. Scenario II: Factors influencing the decision towards admission to or refusal of intensive care

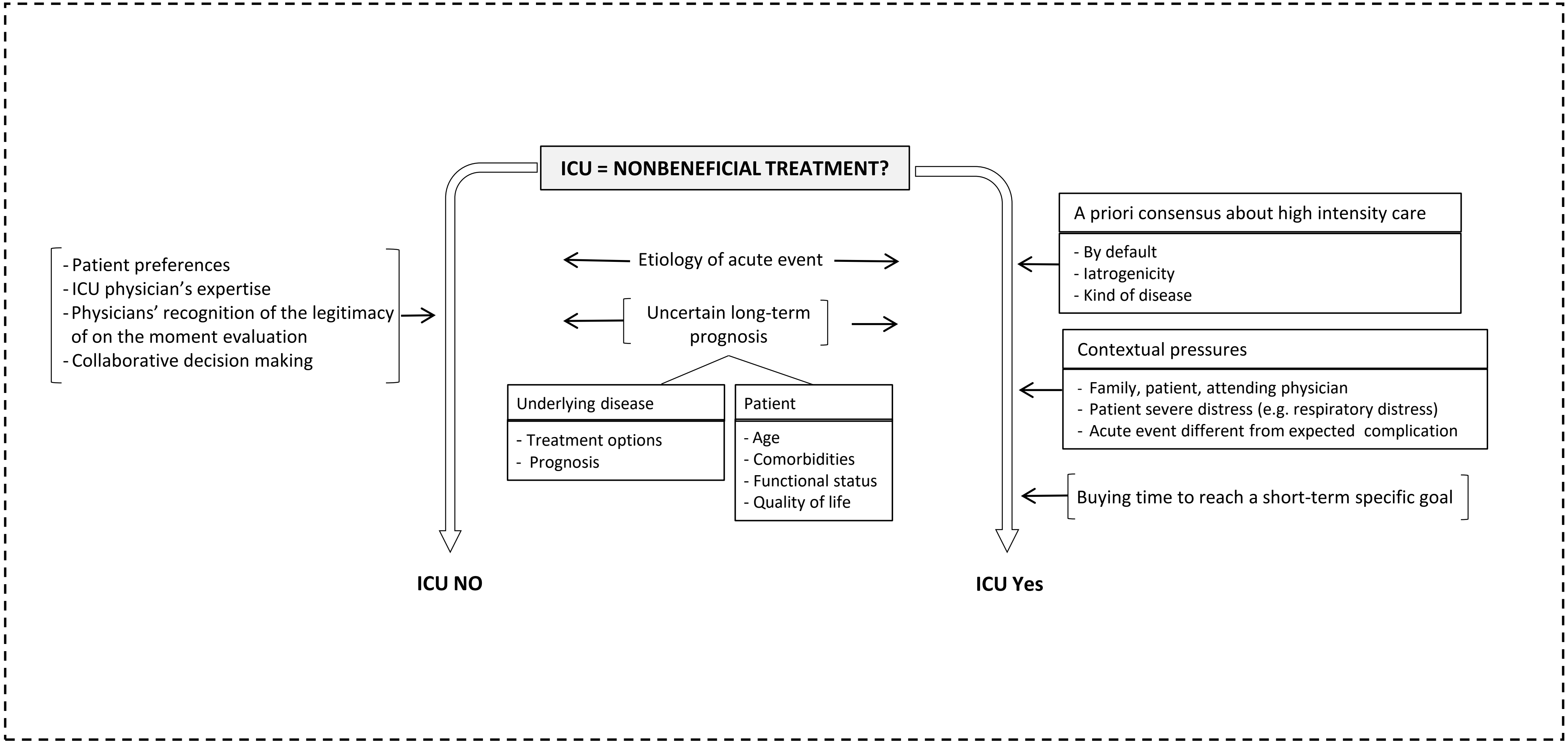

Supplementary file: Interview guide

Please tell me about the first / second situation you have chosen to discuss today.

Prompts:

- Patient characteristics: age? underlying illness? goals of care?
- Context: when dit it occur? what was the reason for calling the ICU? was the patient admitted to the ICU?
- Interactions between the internal medicine and the intensive care physicians:
  - Did the ICU physician come to see the patient?
  - Did you know the other physician?
  - Was the other physician senior or junior to you?
  - What were your expectations with respect to the other physician?

In your opinion, what made the decision-making process easier or more difficult in this situation?

Could you compare the two situations? (after the participant had discussed the two situations)

In your opinion, what is an ideal ICU admission decision-making process?

For peer review only

Table 1 Consolidated criteria for reporting qualitative studies (COREQ): 32-item checklist

No Item	Guide questions/description	
**Domain 1: Research team and reflexivity**		
Personal Characteristics		
1. Interviewer/facilitator	Which author/s conducted the interview or focus group?	**p.10, l.77**
2. Credentials	What were the researcher's credentials? E.g. PhD, MD	**p.10, l.77**
3. Occupation	What was their occupation at the time of the study?	**p.10, l.79**
4. Gender	Was the researcher male or female?	**p.10, l.77**
5. Experience and training	What experience or training did the researcher have?	**p.10, ll.79-81**
Relationship with participants		
6. Relationship established	Was a relationship established prior to study commencement?	**p.10, ll.81-82**
7. Participant knowledge of the interviewer	What did the participants know about the researcher? e.g. personal goals, reasons for doing the research	**p.10, ll.84-85**
8. Interviewer characteristics	What characteristics were reported about the interviewer/facilitator? e.g. Bias, assumptions, reasons and interests in the research topic	**p.10, ll.80-81**
**Domain 2: study design**		
Theoretical framework		
9. Methodological orientation and Theory	What methodological orientation was stated to underpin the study? e.g. grounded theory, discourse analysis, ethnography, phenomenology, content analysis	**p.11, ll.101-102**
Participant selection		
10. Sampling	How were participants selected? e.g. purposive, convenience, consecutive, snowball	**p.9, l.65**
11. Method of approach	How were participants approached? e.g. face-to-face, telephone, mail, email	**p.9, ll.70-75**
12. Sample size	How many participants were in the study?	**p.9, l.66**
13. Non-participation	How many people refused to participate or dropped out? Reasons?	**p.9, ll.74-75**
Setting		
14. Setting of data collection	Where was the data collected? e.g. home, clinic, workplace	**p.10, ll.82-84**
15. Presence of non-participants researchers?	Was anyone else present besides the participants and	**p.10, l.79**
16. Description of sample	What are the important characteristics of the sample? e.g. demographic data, date	**p.9, ll.66-70** **p.13, ll.127-134**
Data collection		
17. Interview guide	Were questions, prompts, guides provided by the authors? Was it pilot tested?	**p.10, ll.76-77**
18. Repeat interviews	Were repeat interviews carried out? If yes, how many?	**p.10, l.96**
19. Audio/visual recording	Did the research use audio or visual recording to collect the data?	**p.10, l.95**
20. Field notes	Were field notes made during and/or after the interview or focus group?	**p.10, ll.95-96**
21. Duration	What was the duration of the interviews or focus group?	**p.10, ll.94-95**

For peer review only

22. Data saturation	Was data saturation discussed?	**p.11, ll.118-119**
23. Transcripts returned	Were transcripts returned to participants for comment and/or correction?	**p.10, ll.96-99**
**Domain 3: analysis and findings**		
Data analysis		
24. Number of data coders	How many data coders coded the data?	**p.11, ll.108-119**
25. Description of the coding tree	Did authors provide a description of the coding tree?	**p.11, ll.111-121**
26. Derivation of themes	Were themes identified in advance or derived from the data?	**p.11, ll.101-103**
27. Software	What software, if applicable, was used to manage the data?	**p.11, l.122**
28. Participant checking	Did participants provide feedback on the findings?	**p.10, ll.96-99**
Reporting		
29. Quotations presented	Were participant quotations presented to illustrate the themes / findings? Was each quotation identified? e.g.participant number	**pp.14-15**
30. Data and findings consistent	Was there consistency between the data presented and the findings?	**yes**
31. Clarity of major themes	Were major themes clearly presented in the findings?	**yes**
32. Clarity of minor themes	Is there a description of diverse cases or discussion of minor themes?	**no minor themes**

For peer review only
